# Characterizing Rigging Crew Proximity to Hazards on Cable Logging Operations Using GNSS-RF: Effect of GNSS Positioning Error on Worker Safety Status

**DOI:** 10.3390/f8100357

**Published:** 2017-09-23

**Authors:** Ann M. Wempe, Robert F. Keefe

**Affiliations:** Forest Operations Research Lab, College of Natural Resources, University of Idaho, 875 Perimeter Drive, Moscow, ID 83844-1133, USA; robk@uidaho.edu

**Keywords:** GNSS-RF, GPS, GNSS positioning error, logging safety, geofences

## Abstract

Logging continues to rank among the most lethal occupations in the United States. Though the hazards associated with fatalities are well-documented and safe distances from hazards is a common theme in safety education, positional relationships between workers and hazards have not been quantified previously. Using GNSS-RF (Global Navigation Satellite System-Radio Frequency) transponders that allow real-time monitoring of personnel, we collected positioning data for rigging crew workers and three common cable logging hazards: a log loader, skyline carriage, and snag. We summarized distances between all ground workers and each hazard on three active operations and estimated the proportion of time crew occupied higher-risk areas, as represented by geofences. We then assessed the extent to which positioning error associated with different stand conditions affected perceived worker safety status by applying error sampled in a separate, controlled field experiment to the operational data. Root mean squared error was estimated at 11.08 m in mature stands and 3.37 m in clearcuts. Simulated error expected for mature stands altered safety status in six of nine treatment combinations, whereas error expected for clearcuts affected only one. Our results show that canopy-associated GNSS error affects real-time geofence safety applications when using single-constellation American Global Positioning System transponders.

## 1. Introduction

Ground workers on cable logging operations work in close proximity to multiple, moving hazards, including highly active heavy equipment, raw materials, and other objects that are swung, dragged, dropped, and dislodged on steep slopes. Proximity to these hazards creates potentially injurious situations for cable logging workers [1–4]. Unlike mechanized, ground-based operations in which employees are generally working within enclosed machine cabs, cable operations rely on ground crew who work unprotected alongside equipment and other hazards in a dynamic environment. Hand fallers and members of the rigging crew face increased risk of injury from hazards such as falling limbs or falling live (green) and dead trees, as well as rolling logs and rocks on steep slopes [1,4,5]. Although United States Occupational Safety and Health Administration (OSHA) and state-level regulations require felling of standing dead trees (snags) within active logging areas [6] (1910.266(h)(1)(vi)), snags may still be present on the periphery of units and during initial work periods prior to felling.

The dangerous nature of logging work is reflected in the industry’s high fatality injury rates, as published annually by the Bureau of Labor Statistics (BLS). The BLS’ 2015 Census of Fatal Occupational Injuries reported 132.7 logger deaths for every 100,000 full-time employees, which was the highest rate of any profession in the United States in 2015 [7]. The rate increased 20% from 2014, when logging also ranked as the most fatal occupation [8]. Lefort et al., who characterized logger injuries in the late 1980s and early 1990s, noted that mechanization of the logging industry had reduced the total number of workplace accidents, but had triggered an increase in injury severity [3]. They tattributed this trend to the changing nature of exposure; ground crew are now working closer to the landing where they face impacts from moving logs and machinery. In 2015, the BLS identified trees, logs, or limbs as the primary source of fatal injury in 41 of 80 total occupational deaths in the logging industry, while 14 deaths were attributed to machinery [9]. Consistent with reports by the Bureau of Labor Statistics, an analysis of W orker’s Compensation claims in West Virginia indicated objects, primarily trees, snags, or logs, striking crew members accounted for 47% of injuries, more than any other cause [1]. Similarly, according to claims records from eight southern states in 1997, falling trees or limbs and moving logs caused the most accidents (28% of injuries), followed by equipment, including skidders, feller-bunchers, dozers, and loaders (23%) [4].

GNSS-RF (Global Navigation Satellite System-Radio Frequency) transponders have the potential to reduce the incidence of injuries and fatalities on logging operations by improving situational awareness. GNSS-RF units determine their coordinates from one or more navigation satellite systems, including the United States’ Global Positioning System (GPS), Russia’s Global Navigation Satellite System (GLONASS), China’s BeiDou, or Europe’s Galileo. They then transmit those coordinates to other units locally by data transfer using radio frequency transmission. Used in conjunction with mobile devices such as phones or handheld tablets, or onboard computers, GNSS-RF technology can provide a real-time, systemic visualization of all the interacting components of logging operations, supplementing voice communications used conventionally on two-way radios and signal horns such as Talkie-Tooters (Rothenbuhler Engineering, Sedro Woolley, WA, USA). With knowledge of ground crew positions in relation to potential hazards, machine operators could make more informed decisions based on the known locations of workers displayed on maps on mobile devices and, in some cases, supplement the use of conventional audible communication with visual or audible alerts indicating worker presence in work zones delineated by geofences [10–12].

GNSS has been utilized widely in forestry for decades. GNSS is integrated into Geographic Information Systems (GIS) to map ownerships and delineate stand boundaries, forest road locations, and other features on timber sales [13]. Mobile positioning devices have been installed on harvesting machines to track movement over the course of harvest operations and assess soil impacts and performance [14,15]. GNSS is increasingly being used in place of traditional, observational methods to characterize productive cycle-times of forest machines [16–18]. Harvesters have been fitted with GNSS devices to collect tree positioning data [19,20]. Development of GNSS paired with RF for real-time positioning is emerging quickly in forestry and has a variety of potential uses including operational and wildland fire logistics, real-time optimization, and safety [10–12,16].

Situational awareness can be augmented further by combining GNSS-RF positioning with virtual boundaries known as geofences, which delineate hazardous areas, silvicultural treatments, or work zones on timber sales [10,11]. Geofences provide a means by which to monitor the current locations of people, equipment, or other resources relative to spatial boundaries and can be programmed to alert users of crossing events [21]. They have been successfully integrated into various industries to help resolve positional monitoring and restriction needs [21–26] and have potential applications in logging to alert machine operators about ground-worker proximity [10–12].

To improve logging safety, geofence boundaries need to account for dynamic positional relationships between workers and hazards. As ground workers move throughout cable corridors, spatial proximity of people to one more pieces of equipment, snags, skyline rigging, and harvested resources are in constant flux. OSHA, which establishes guidelines and regulations for safe practices on logging operations in the United States, does not provide explicit safe distance recommendations for most logging equipment. Rather, it relies upon workers to interpret safe proximity in situational context. OSHA regulation 1910.266(f)(2)(vii) states that a “machine shall be operated at such a distance from employees and other machines such that operation will not create a hazard for an employee” [6]. Oregon OSHA Division 7 (2009), as well as common industrial safety awareness campaigns, advise workers to stay “in the clear”, which generally is translated as a distance equivalent to the length of a tree or log being transported to the landing [27]. However, if loggers frequently occupy areas less than one tree length from a hazard, the geofence associated with that hazard may need to be smaller than the recommended safety distance in order for operators to discern between normal activity and higher risk situations, or early warning signals may need to be deployed. Knowledge of positional relationships will also help define GNSS accuracy needs. If ground crew generally work within 5–10 m of a hazard, positioning errors greater than 5 m may be detrimental to safety; whereas lower accuracies may still be useful in improving general awareness if workers already avoid proximity to hazardous areas. The use of mobile geofences, which can move with hazards, introduces additional considerations, such as geofence alert accuracies associated with the geometry of multiple moving components [12].

Although the sources of occupational injuries and fatalities are well-documented for logging and use of geofences for logging safety applications has been studied in designed experiments, spatial analysis of the actual positional relationships between workers and some common hazards on active operations has not been quantified or summarized previously. In fact, despite the widespread attention to spatial proximity in safety training as well as state and federal regulations in forestry, there has been virtually no prior analysis of actual positional movements among ground workers of the sort that is now possible using GNSS-RF technology. In this paper, we characterized the real-time positions of ground crew workers and three common situational hazards during active cable operations using coordinates collected by Raveon Atlas PT GNSS-RF devices (Raveon Technologies Corp, San Diego, CA, USA), which feature a VHF data modem combined with a 12-channel GNSS receiver that receives position information from a single constellation, the American NAVSTAR GPS system. It is important to note that the devices do not receive positional information from GLONASS, BeiDou, or Galileo, as some other current GNSS-RF devices do. We summarized safe worker-hazard distances by calculating the amount of time, in one second increments, that workers occupied zones outside (“safe”) and inside (“unsafe”) circular geofence boundaries assigned to each hazard. Because forest overstory is known to impact GNSS accuracy [28–30], we also conducted a designed experiment on the University of Idaho Experimental Forest to quantify canopy impacts on receiver accuracy in both mature and recently clearcut stands. These conditions correspond to the early stages of harvesting operations (canopy intact), transitioning to later stages (canopy removed) that result during typical clearcut operations in the northwestern United States. We then used simulation to re-analyze our operational data, in order to evaluate the extent to which canopy-induced error, as determined in the earlier designed experiment, affected the GNSS-characterized safety status of ground workers over the course of active operations.

Our specific objectives were to determine whether the proportion of unsafe time, defined as time spent inside one or more hazard geofences, differed by (1) hazard type (loader, carriage, snag); (2) timber sale; or (3) GNSS environment (observed, mature, clearcut).

## 2. Materials and Methods

### 2.1. Controlled Experiment

#### 2.1.1. Data Collection

To estimate the impact of GNSS error on operational positioning data collected at logging operations, we first calculated Atlas PT error in a controlled experiment on the University of Idaho Experimental Forest (UIEF) in Princeton, Idaho (USA). The UIEF encompasses canopy features and slopes representative of north Idaho mixed-conifer forests, ranging in age from recent clearcuts to mature stands approximately 90 years old. Eight stands were selected for sampling in the Flat Creek and East Hatter units of the UIEF, all located at mid-elevation (approximately 915 m) on the north slope of Moscow Mountain in the Palouse Range, in the vicinity of 46.8413° latitude, −116.7734° longitude. Four stands were clearcut harvested within 5 years prior to the experiment, which took place in October and November of 2016. The other four stands were over-mature, with most trees approximately 80–90 years old, having regenerated after railroad logging in the early 20th century. Based upon plot inventories completed in each stand following sampling, tree heights ranged from 3.2 to 40.3 m in mature sites (mean of 18.2 m), and diameters at breast height (DBH) ranged from 13 to 89 cm (mean of 31 cm). Stands were comprised of ponderosa pine *(Pinus ponderosa)*, grand fir *(Abies grandis)*, western larch *(Larix occidentalis)*, western white pine *(Pinus monticola)*, Douglas-fir *(Pseudotsuga menziesii)*, and western red cedar *(Thuja plicata)*. Table 1 shows stand characteristics measured at time of sampling (azimuth, slope) and during inventory (height, DBH), as well as sampling conditions, including satellite availability and constellation. The number of in-view satellites was recorded every second by each of four Atlas PT transponders during sampling and then averaged across all devices. A Differential GPS (DGPS) GNSS receiver, the Arrow 100 made by EOS Positioning Systems (Terrebonne, QC, Canada), collected position dilution of precision (PDOP) values at each Atlas PT location. Low PDOP values (less than 4) indicate lower GNSS positioning error and are a function of satellite constellation orientation.

**Figure 1.**
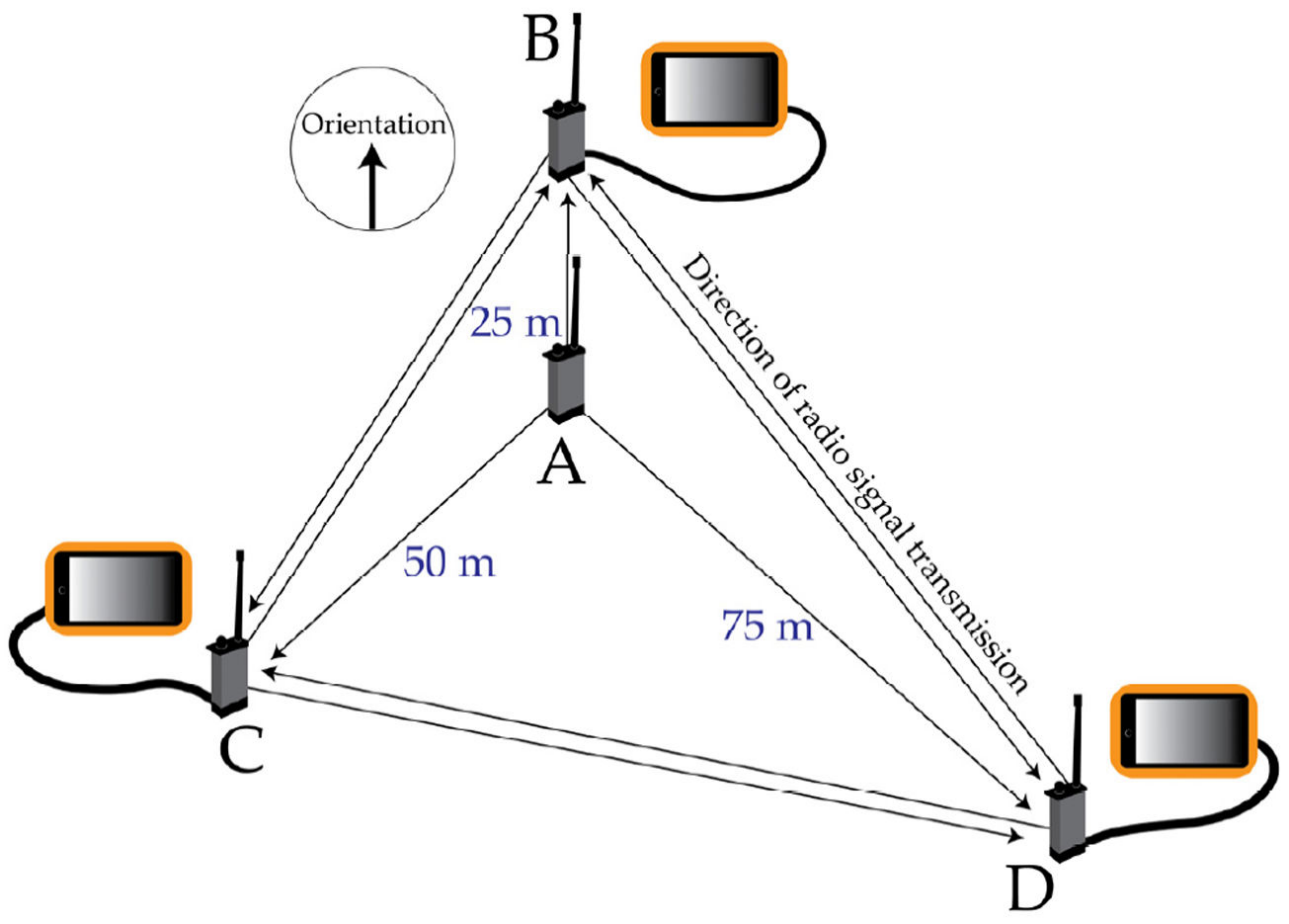
Design of the controlled experiment.

At each stand, we collected GNSS positioning data using four Atlas PT transpon units. We sampled for thirty minutes at a transmission frequency of one second, allowing for a potential of 1800 total observations per unit per site (actual signal transmission efficiency ranged from 0.739 to 0.997). Each unit was fastened to a wooden post using plastic zip ties, such that the base of the radio antenna was positioned at a height of one meter above the ground. The Atlas PTs were arranged in a triangular plot as shown in Figure 1, with a centrally located unit (“A “) positioned 25 m in slope-distance from unit “B”, 50 m from “C “, and 75 m from “D”. The orientation (azimuth) of unit B from A was selected randomly prior to sampling, and orientations for C and D were measured using a Suunto azimuth compass at 120° (±0.5°) from unit B’s orientation.

During sampling, each Atlas PT transmitted its location coordinates to each other unit once per second via radio frequency. Units; B, C, and D were connected to Dell Venue Pro 8 5855 (Dell Inc., Round Rock, TX, USA) tablets equipped with Raveon RavTrack PC real-time tracking software (Version 6.5,2015, Raveon Technologies Corp, San Diego, CA, USA) and Microsoft Access™ (Version 16.0,2016, Microsoft, Redmond, WA, USA). Tablets automatically logged transmissions to an MS Access database for subsequent analysis. The same Atlas PT and tablet were used for each position (A, B, C, D) at every stand.

#### 2.1.2. Estimation of Error

To determine the positioning error associated with Atlas PTs, we compared the recorded (observed) coordinates to reference coordinates determined in real-time using the Arrow 100. The Atlas PTs receive single frequency (L1) signals from the United States GPS system and are capable of static horizontal accuracies of less than 2.5 m 50% of the time and less than 5 m 90% of the time. The Arrow 100 (also single frequency) is a multi-constellation receiver utilizing GLONASS and BeiDou in addition to GPS. Differential correction with a Satellite Based Augmentation System (SBAS), which is the Wide Area Augmentation System (WAAS) in the United States, enables it to achieve accuracies less than 60 cm. After the four Atlas PTs were situated in the arrangement described above, the Arrow 100 was placed at each Atlas PT position where it recorded coordinates and position dilution of precision (PDOP) values (see Table 1). After sampling, we projected all data to the Universal Transverse Mercator (UTM) projection, which has units in meters, using ArcGIS 10.3 software (Version 10.3.1,2015, Esri, Redlands, CA, USA). We then calculated the horizontal error for each observation (each 1 second-interval transmission) as the hypotenuse distance between the two sets of UTM easting (denoted *UTMe)* and UTM northing (denoted *UTMn)* points (actual—observed), as shown in Equation (1). Observed coordinates were retrieved from unit B’s transmission log of all four Atlas PT positions over the sample period.

(1) $$Error\;=\;e\;=\;\sqrt{{\left(act.UTM_{e}\;-\;obs.UTM_{e}\right)}^{2}\;+\;{\left(act.UTM_{n}\;-\;obs.UTM_{n}\right)}^{2}}$$

We determined if this error varied by stand, cover, or individual transponder unit using an Analysis of Variance (ANOVA), and then we identified significant sources of variation among stands and transponders using a Bonferroni multiple comparison test.

Error was summarized for each unit and each stand as the root mean square error (RMSE), which is a measure of the difference between predicted (based on the Arrow 100) and observed (based on the Atlas PT) values.

(2) $$RMSE\;=\;\sqrt{\frac{\sum \;e^{2}}{n}}$$

where *e* represents error as calculated in Equation (1), and *n* represents the sample size (number of 1-second transmissions). All calculations and statistical analyses for the study were completed using R open source statistical computing software (Version 3.3.1,2016, The R Foundation, Vienna, Austria) and are presented in the Results section [31].

### 2.2. Operational Sampling

#### 2.2.1. Data Collection

**Figure 2.**
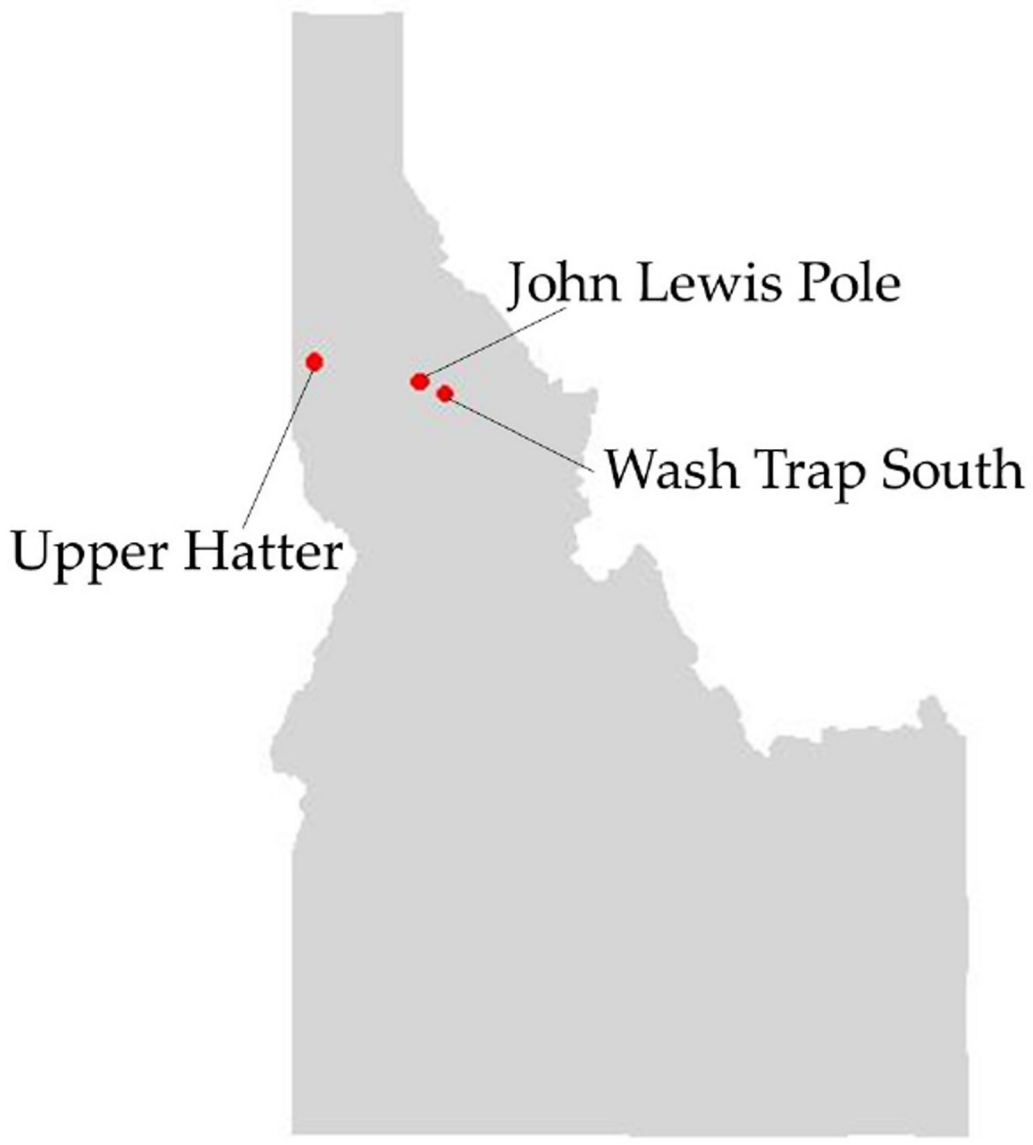
Idaho state neap, with logging operation sites shown in red.

GNSS positioning data were collected using Atlas PT units at three active cable logging operations in north Idaho (see Figure 2) on slopes ranging from 40–65%. All logging activities were conducted by professional, certified logging contractors on regular, operational timber sales at three ownerships: Idaho Department of Lands state endowment land (John Lewis Pole, or JLP), Potlatch Corp (Wash Trap South, or WTS) and the University of Idaho Experimental Forest (Upper Hatter, or UH). All operations were rigged for uphill yarding using motorized carriages. The state and industrial operations had swing yarders (Linkbelt 90 and Skagit GT-4, respectively) and the contractor working on the UIEF had a custom excaliner constructed on a John Deere carrier. Ground crew were responsible for setting chokers (the hooker, in regional terminology) and unhooking chokers when logs reached the landing (the chaser). The W TS and UH timber sales were clearcut operations, while the state JLP timber sale was a cedar pole harvest. Under Idaho law, cedar poles are required to be removed prior to other harvesting on cable operations when more than 10 poles per acre are present.

**Figure 3.**
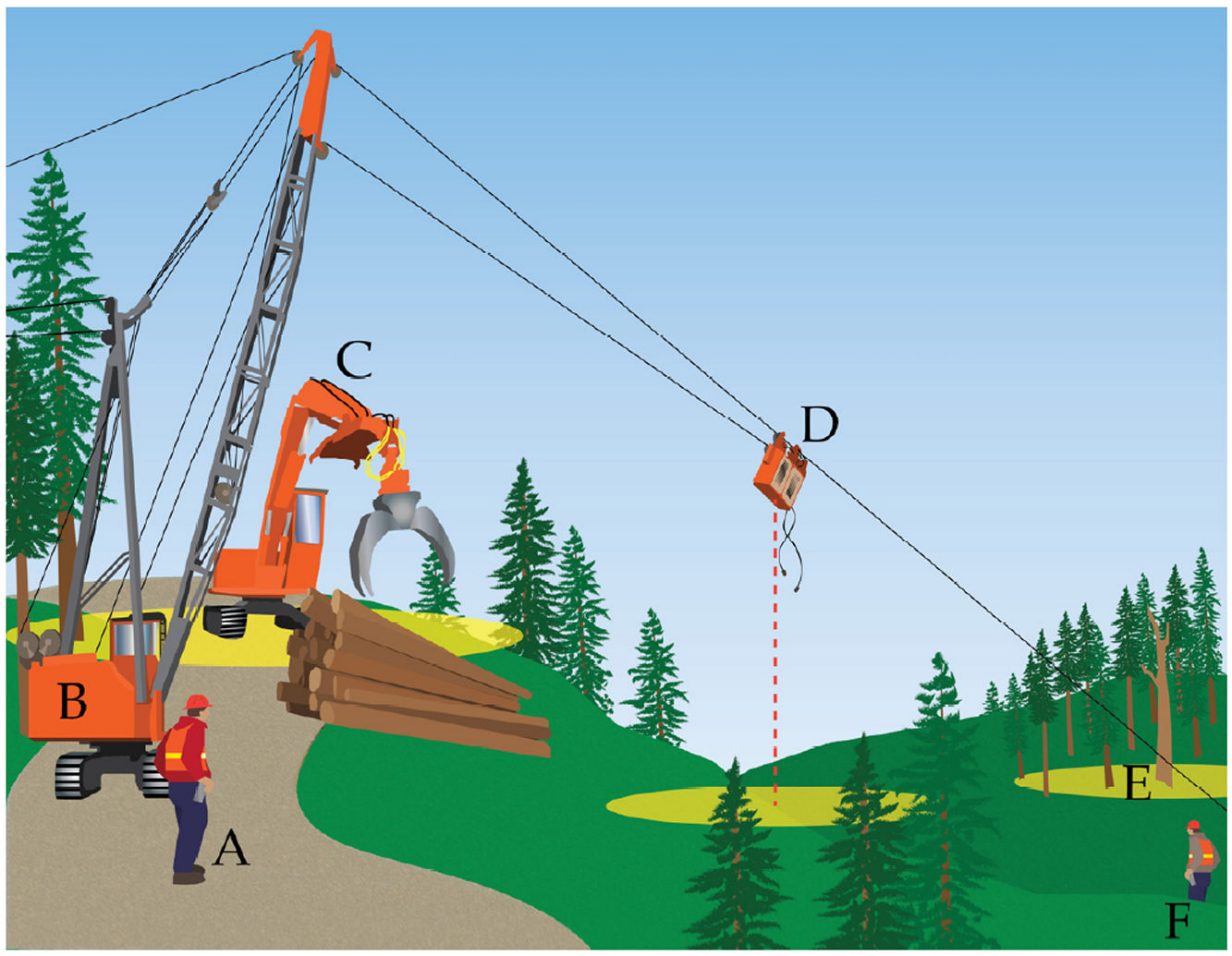
Typical cable logging operation wish **(A)** chaser; **(B)** yarder; **(C)** loader; **(D)** carriage; **(E)** snag; and **(F)** hooker (note: images are not drawn to scale). Yellow ellipses highlight areas with increased risk of injury associated with the three types of hazards shown. Of the three hazards, snag GNSS coordinates were fixed (static), while loader and carriage locations were dynamic.

Excludingroad (corridor) repositioning and equipment rep airs, at least ten hours of positioning data were collected during regular operations at each harvest over the course of three days per site, with sampling; occurring between mid-August and December 2 016. Worker Atlas PT units were placed in radio pouches for protection and then distributed to ground crew, who wore the radio pouches on their belts. Machine-mounted units were attached to the exteenal, metal grating covering cab windows, where the unit antennas (GPS and radio) were unobstructed. Skyline caroiage units were secured to the carriage top using zip ties, such that antennas were exposed to the sky. At each site, an Atlas PT placed uphill on the yarder cab was connected to a Dell tablet for data collection and real-time visualization of unit positions with Raveon RavTrack software using the methods as in the controlled field experiment. In addition to collecting fluid GNSS coordinates of mobile hazards with the Atlas PT units, we also identified a snag or danger tree within the harvest unit and recorded its location with a Garmin 64 handheld GPS (Garmin Ltd., Olathe, KS, USA). Thus, our raw delta comprised of GNSS positioning for two to three gtound workers and three types of operational hazards: machinery (the loader), equipment (the carriage), and a stationary, environmental hazard (the snag) (see Figure 3). After sampling, coordinates from the Atlas PTs and Garmin were converted to UTM for all analyses.

#### 2.2.2. Summarizing Worker Proximity to Hazards

We defined worker safety in terms of worker position relative to circular geofences surrounding each of the three hazards. Conceptually, the area inside each geofence was assumed to represent a work area with higher risk of fatal or non-fatal traumatic injury due to the potential for being struck by the hazard. While work inside these areas is necessary on partially mechanized operations, the presence of ground workers within geofenced areas require increased situational awareness and caution on the part of both ground workers and operators to avoid accidents. Areas beyond geofence borders encompass safe work areas, where the risk of injury from striking hazards is generally lower. Thus, at any given time, a worker was positioned either inside or outside the hazard geofences of 0–3 hazards and was classified as either safe or unsafe relative to each. Similarly, at any time, a given hazard such as the skyline carriage might have as many as three rigging crew workers in close proximity.

Using the statistical programming environment, R, we created geofences centered at each hazard’s coordinates, as recorded by the associated Atlas PT (or Garmin, in the case of the snag). The carriage geofence was assigned a radius of 30 m, approximating one tree length, to encompass the risk of being struck by both the carriage itself, swinging choker cables, or logs being yarded by the carriage. The loader geofence also had a radius of 30 m (one tree length). The snag’s geofence radius of 60 m represented two tree lengths, which is the standard recommended safe working distance from danger trees published by OSHA [6] (1910.266(h)(1)(vi)).

For each one second time stamp in the operational data, we calculated the distance of all ground crew from each of the three hazards. For the purposes of spatial analysis, and in order to summarize 6–7 entities moving dynamically in time and space, we grouped proximities in 5 m increments from 0–350 m. We then summed the frequency at which workers occupied each proximity zone at each of the three timber sales, as well as the proportion of time spent in safe and unsafe zones, internal and external to the geofence associated with each hazard. To simplify analysis, ground workers were analyzed as a group rather than individually. Rigging crew workers in the region regularly alternate roles, switching among, for example, hooking and chasing, and thus summarizing across all work tasks allowed workers to retain the same Atlas PT units without stopping to switch. Also, for the purposes of analysis, the locations of the workers and hazards reported by Atlas PT GNSS positioning were considered observed coordinates with an expected degree of error comparable to the error evaluated previously in the controlled experiment.

We used the Marascuillo Procedure for comparing multiple proportions to test the null hypotheses that the proportion of observed worker presence in unsafe areas did not differ by (1) timber sale or (2) hazard type. The Marascuillo Procedure compares the test statistic (see Equation (3)) to a critical value (Equation (4)) calculated for each pair of proportions in a way that accounts for degrees of freedom when comparing multiple proportions simultaneously.

(3) $$value\;=\;\left|p_{i}\;-\;p_{j}\right|$$

(4) $$critical\;range\;=\;r_{ij}\;=\;\sqrt{X_{1-\alpha ,\;\kappa -1}^{2}}\;\sqrt{\frac{p_{i}(1-p_{i})}{n_{i}}+\;\frac{p_{j}(1-p_{j})}{n_{j}}},$$

where X^2^1−*α,k−1* is the chi-square distribution with a confidence interval of 1 – *α (α* is the significance level) and degrees of freedom equal to 1 – *k*, (*k* equals the number of populations). *pi* represents the proportion for sample *i*, *pj* represents the proportion for sample *j*, and *n* represents the sample size. If the value from Equation (3) is greater than the critical range, then the two compared proportions are significantly different.

#### 2.2.3. Simulation of GNSS Error

A simulation script was written in the R language in order to assess the effect of horizontal positioning error on the safety status of individuals. For each one second time stamp in the operational data, a one second observation was selected at random from one of the four mature or clearcut plots in the controlled experiment described previously. We applied an error adjustment to the operational data based upon the UTM easting and UTM northing differences, as well as azimuth (in degrees) from the actual (Arrow 100) and observed (Atlas PT) coordinates. Thus, we assumed for the purposes of analysis that each worker location in the operational data was uncorrected, and then shifted each coordinate individually by a distance and direction corresponding to either mature canopy or clearcut error accuracy from the controlled experiment. To simplify analysis, we assumed that hazard locations were true coordinates; thus, they were not adjusted during simulation. 500 iterations of the simulation script were processed. After resampling and application of error adjustments to worker positions, inter-point distances from each of the three jobsite hazards were again summarized in zones of 5 m increments, and the proportions of safe and unsafe status were determined. Since adjustments to the operational data were sampled from individual GNSS errors recorded on multiple different sites and dates, simulated data do not represent the true location of each worker at a given time. Rather, we utilized simulation to provide an indication of the degree of impact to be expected from positioning error in relation to a fixed point (the geofence) in each GNSS environment (observed, mature, or clearcut).

We determined whether GNSS positioning error would impact definitions of workers as safe or unsafe based on proportions of time spent inside geofenced hazard zones. Using the Marascuillo Procedure, we tested the null hypothesis that unsafe proportions were equal for observed, mature, and clearcut data for each hazard at each site, where observed data represented worker positions as recorded by the Atlas PTs, mature data represented simulated worker positions accounting for GNSS error associated with canopy, and clearcut data represented simulated worker positions accounting for GNSS error under un-obstructed conditions. The Marascuillo Procedure was performed for each iteration, and the mean value was compared to the mean critical range to determine if proportions differed significantly.

## 3. Results

### 3.1. Controlled Experiment: Estimating Atlas PT Positioning Error

Plot-level Root Mean Squared Error (RMSE) calculated for all four Atlas PT units within each stand (Equation (2)) ranged from 2.64 m to 4.09 m in clearcuts, with the best accuracy achieved in Stand 531. By contrast, RMSE ranged from 8.56 m to 14.34 m in mature stands, with the lowest accuracy occurring in Stand 524 (see Table 2 for RMSE by stand and unit). The RMSE of all mature stands combined was 11.08 m, while the overall RMSE of clearcuts was 3.37 m. With the exception of one unit (B in Stand 58), RMSE values in clearcut conditions are under the 5 m accuracy expected for Atlas PTs 90% of the time. However, none of the devices in mature stands achieved this level of accuracy.

**Figure 4.**
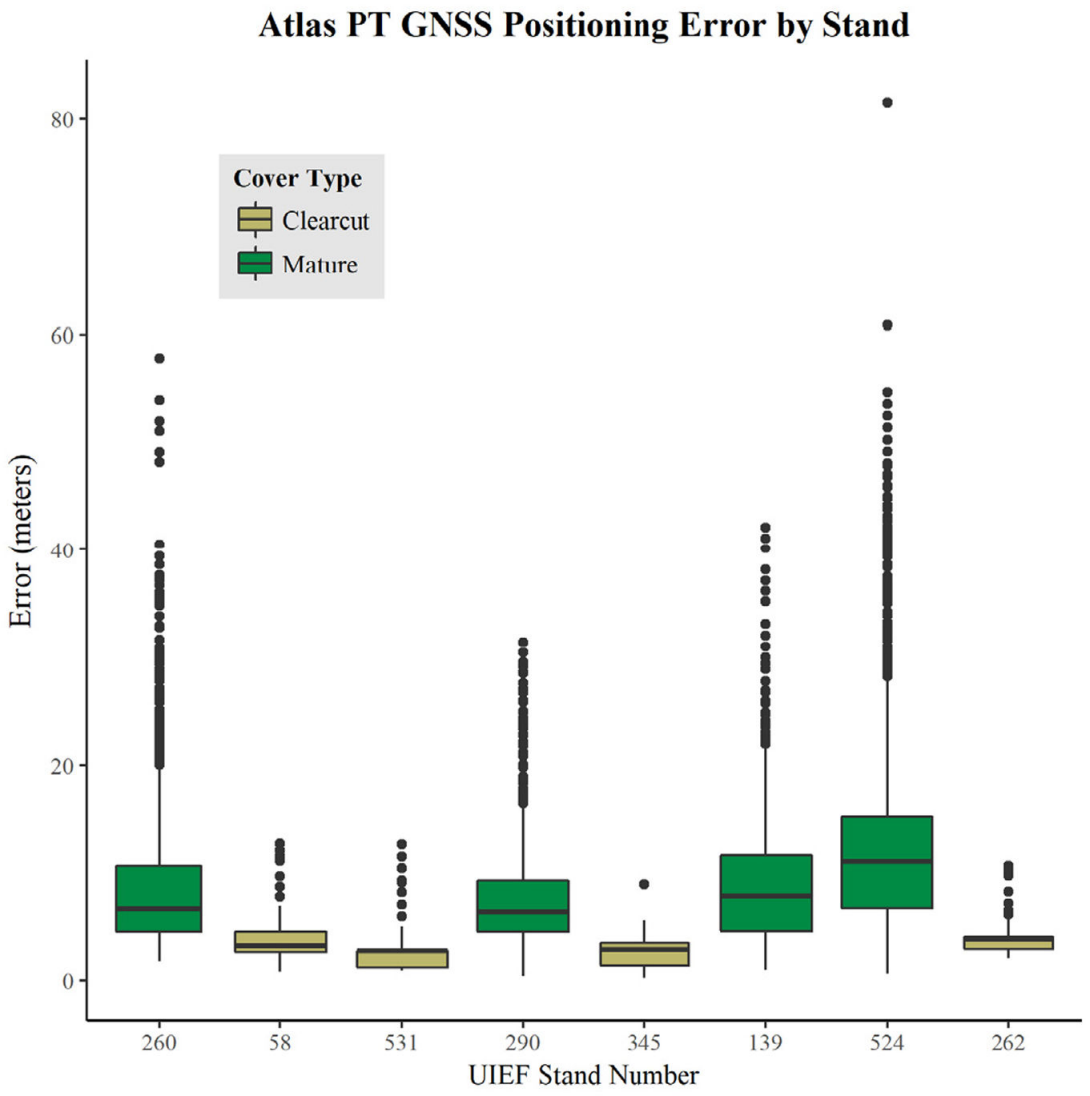
Boxplot comparing Atlas PT actual GNSS error in the controlled experiment across eight stands: four mature (green) and four clearcut (tan).

Actual error calculated for each second of sampling (Equation (1)) varied significantly by stand (F-statistic = 4735, *p*-value < 2 × 10^−16^), cover (F-statistic = 25,390, *p*-value < 2 × 10^−16^), and individual transponder unit (F-statistic = 337.3, *p*-value < 2 × 10^−16^). The Bonferroni multiple comparison test comparing all stands indicated that only two stands did not differ significantly from one another (clearcut units 345 and 531, with *p* = 1.00). Multiple comparison indicated that all transponder units differed significantly from one other (*p*-values less than 2 × 10^−16^) except for units B and D (*p* = 0.089). Figure 4 illustrates actual error variation across stands of different cover types.

**Figure 5.**
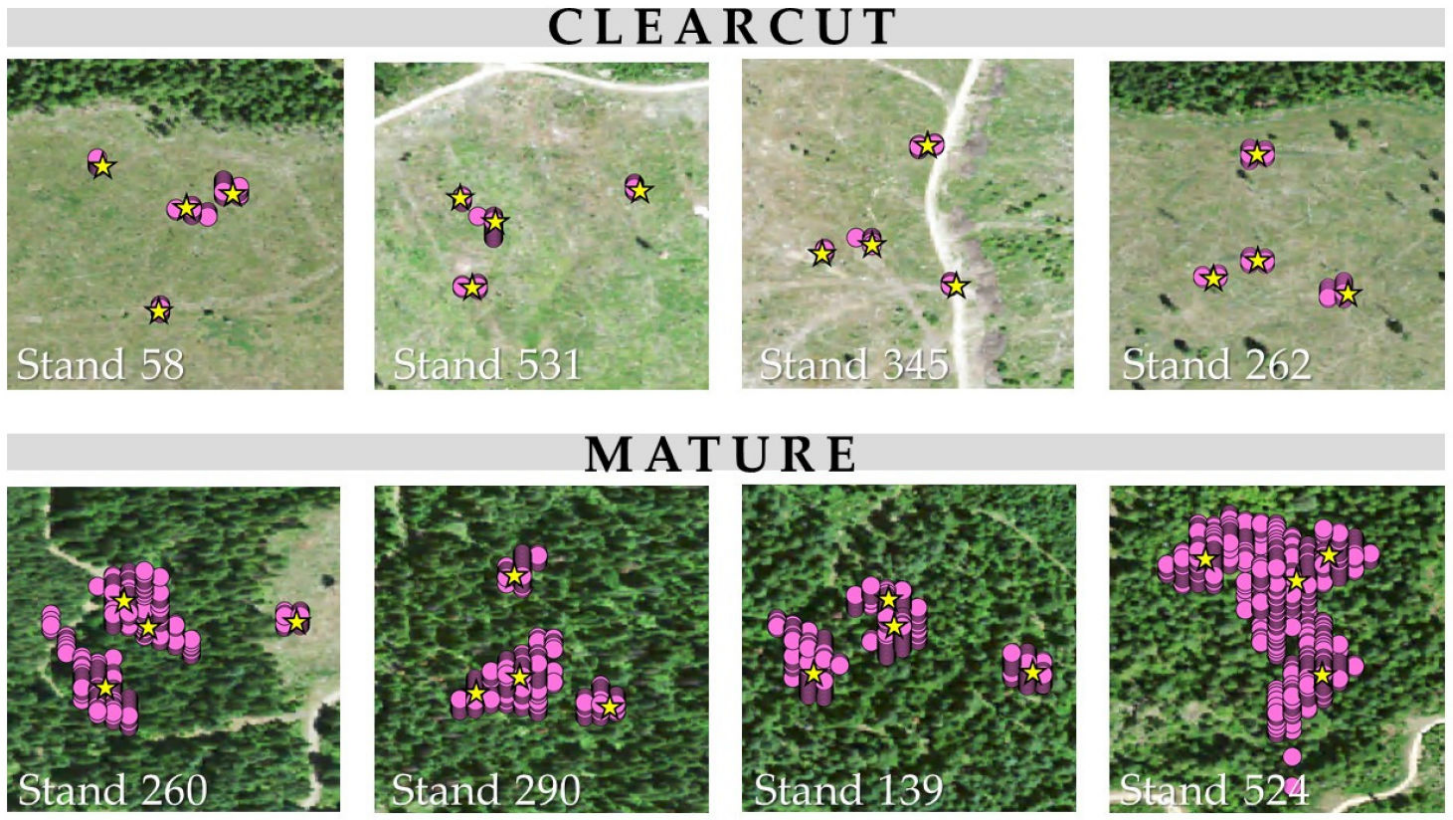
Visual comparison csl: Atlas. PT coordinates (purple dots) and Arrow 100 coordinates (yellow stars) in clearcut versus mature stands of ths controlled experiment. Scale i s 1:1500.

Figure 5 illustrates the distribution of Atlas PT GNSS posieions coliected over each 30-min sampling period compared to the single coordinates recorded by the EO SA rrow 100 at (each Atlas PT location. The largest actual error observed for an Atlas PT in a mature stand was 81.5 m, and the largest error observed in a clearcut stand was 12.6 m.

### 3.2. Operational Sampling: Summarizing Worker Positional Relationships to Hazards

**Figure 6.**
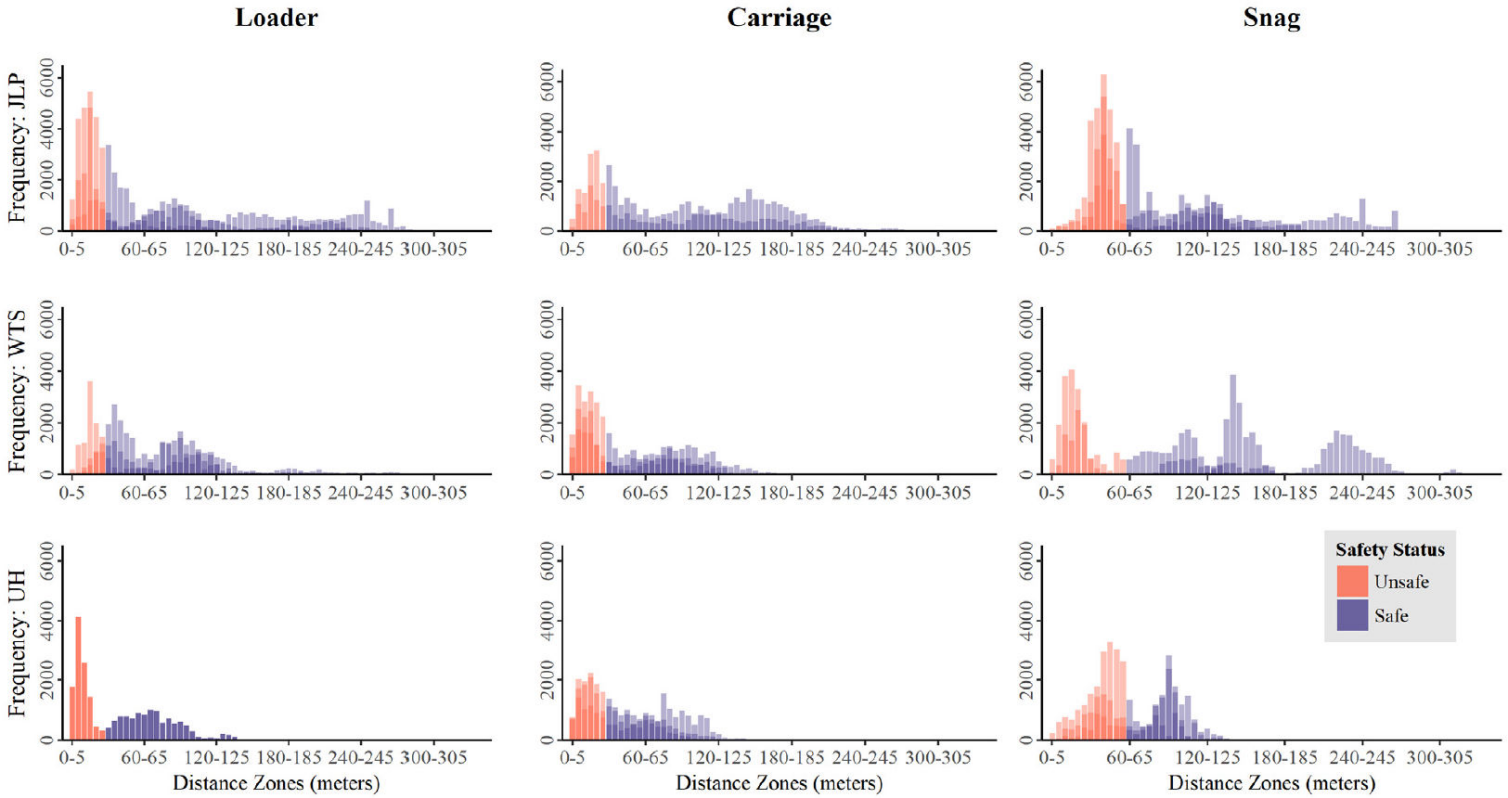
Distribution of ground worker distances from each of three hazards (loader, carriage, and snag) across three logging operations (John Lewis Pole, Wash Trap South, and Upper Hatter). Vertical bars are in 5 m increments. Red bars indicate location inside of the hazard’s geofence (Safe); blue bars indicate location outside of the geofence (Unsafe). Shades of color represent different sampling dates.

Figure 6 illustrates the distribution of worker-hazard distances for each of the three hazards at each harvesting operation. Bars show frequency of ground worker presence within distance zones in increments of 5 m, ranging from 0 to 350 m from the specified hazard. John Lewis Pole plots represent three ground workers (the chaser, bucker, and hooker), while Wash Trap South and Upper Hatter encompass positioning data of two workers (the chaser and hooker). Each of three days is overlaid for a given site and hazard, except for the John Lewis Pole Carriage, which included two days of sampling, and Upper Hatter Loader, which included one day. Distances are based on GNSS coordinates collected every one second (s); thus, a frequency of 6000 corresponds to 6000 s (100 min) sp ent insnide a given proximity interval. The proportion of time in which ground crew occupied zones defined as unsafe due to increased risk of injury os fatality is summarized in Table 3. Unsafe zones were defined as distances between 0–30 no for tire loader and carriage and 0–60 m for the snag. Observed (Obs.) values are based upon GNSS positions collected by Atlas PTs during sampling and subsequent calculations of distances from leazards in R. Mature (Mat.) and clearcut (Clear.) values represent mean values for the GNSS environment simulated with and without mature forest oveastory across 500 iterations applied to observed data based on sampled horizontal positioning error as measured in the controlled experiment. Proportions cover three sampling days at each cur three sites: John Lewis Pole (J LP), Wash Trap South (WTS), and Upper Hatter (UH). Sample sizes are indicated in parentheses below each set of proportions. Differences in sample sizes eeflect misaing GNSS coordinates Sar a hazard, either due to poeiUoning or transmission eroor, or because the eqrnpment designated ess a hazard was not in operation foe a portion of the sampling period. Proportions shown in Table 3 were used in the Marascuillo Procedure analyais.

Across all days and all sites, ground workers spent a combined 18.5 hours (h) within 30 m of the loader geofence (34.6% of their time), 21.4 h within 30 m ob the carriage (38.7% of time), and 32.3 h within 60 m of the snag (46.7%). It is important to note that our arsults represent collective ground worker positioning da0a, pooling the chaser and hookea (as well as a bucker for John Lewis Pole). Chasers generahy wark close to the landing1 while hookers work varying distances along the cable corridor, so proximity to landing hazaads such as the loader would be expected to differ for the two workers.

Results from the Marascuillo Procedure comparing proportions among hazards and sites are shown in Table 4. For each pair of comparisons, the table shows the value (Equation (3)) representing the Marascuillo test statistic and the critical range (Equation (4)). If the value exceeds the critical range (“yes”), the difference in the two compared proportions is significant. The Marascuillo Procedure compared 36 total proportions but only the 18 tests of interest in our study are shown. They include comparing (1) each hazard across all three sites: Loader (Tests 3, 4, and 12), Carriage (6, 7, and 14), and Snag (8, 9, and 15) and (2) each site for all three hazard types: John Lewis Pole (1, 2, and 5), Wash Trap South (10,11, and 13), and Upper Hatter (16,17, and 18). All 18 hazard and site comparisons of interest were significant, indicating that the proportion of worker presence inside geofence boundaries varied by hazard type and site.

Results of the Marascuillo Procedure comparing unsafe proportions between observed, operational data with and without simulated canopy and clearcut error effects are shown in Table 5. The table summarizes the results for nine separate tests, each with three comparisons in which observed (Obs.), mature (Mat.), and clearcut (Clear.) proportions (see Table 3) were compared for a single hazard at each timber harvest. Observed proportions differed significantly from mature proportions for six of nine hazards: JLP Loader, JLP Carriage, JLP Snag, W TS Carriage, UH Carriage, and UH Snag, but differed significantly for only one clearcut proportion (JLP Snag). Mature and clearcut proportions differed significantly from each other for six of nine hazards: JLP Loader, JLP Carriage, JLP Snag, WTS Carriage, UH Carriage, and UH Snag.

## 4. Discussion

Our results showed clearly that the nature of positional relationships was complex and varied both between sites and between hazard types in each treatment comparison tested. Distinct, multi-modal patterns of worker proximity to hazards were evident, and the locations of peak distances where workers tended to spend more time varied by day. Although we did not formally test differences among the three days sampled at each site, it was evident graphically when overlaying the distributions of proximity (Figure 6) that distinct patterns of spatial proximity exist and change over time. These trends likely correspond to, for example, hookers gradually working further from the loader as they set chokers and yard materials to the landing from further down the hill, or gradually working either closer to or further away from snag hazards identified adjacent to the harvest units.

A more nuanced analysis of individual worker positions relative to multiple hazards, such as studying hooker or chaser movements separately, could help to better quantify the spatial and temporal nature of positional relationships during normal work. However, we felt that our analysis reflected the reality of cable logging, in which multiple hazards are present simultaneously for any given worker, often in different directions. For example, a member of the rigging crew setting chokers near the top of the hill may be at risk of impact from rolling logs inadvertently bumped by the loader at the log deck. At the same time, he or she may also be at risk of being hit by a rotating log attached to a choker as the carriage begins to laterally yard logs toward the skyline if not sufficiently ‘in the clear’ at a safe distance horizontally across the hillslope from the carriage (and log). Simultaneously, a snag on the perimeter of the corridor could fall if dislodged by the log being yarded or a cable under tension. Although we focused on three possible hazards, the reality of cable logging is that multiple, other concerns are also present, including the processor swinging logs, pinch points caused as swing yarders, loaders, and processors rotate adjacent to the cut slope of the logging road, possible chain shot from the processor, and loose boulders in the corridor that may become dislodged.

Results of our controlled experiment on the UI Experimental Forest showed that the positioning accuracy of the GNSS-RF transponders used in our study was greatly affected by canopy. RMSE for the Atlas PT GNSS receivers in clearcuts was 3.37 m; whereas in mature, 90-year old mixed conifer stands, the RMSE was 11.08 m. The error observed in our study represents a function of variables affecting positioning accuracy, including Atlas PT receiver quality, the satellite geometry for the specific times and dates of sampling (see Table 1 for PDOP values), and multipath effects unique to the individual environments of each site. Improved GNSS receivers may demonstrate higher accuracies, even in mature stands. GNSS error associated with forest canopy has been well-documented though [28–30], so the observed variation in positioning accuracy by cover type is consistent with past studies.

When simulation was used to evaluate the relative importance of variable GNSS accuracy on worker safety status during active logging, results clearly showed that canopy-induced error did significantly affect the safety status, as defined using geofences. It is important to note that simulated canopy and clearcut error impacts on worker safety status were based on resampling from positioning data obtained at different locations, dates, and times than the operational sampling, so our results serve as an approximate estimation of canopy effects; actual error observed at active logging operations may differ due to topography or other factors. Further, error estimates based on static positioning in the controlled experiment were likely more conservative than error associated with dynamic positioning during active logging operations [32,33]. A further caveat we wish to highlight is that the statistical method used in our analysis to evaluate differences among sites and hazards, the Marascuillo Procedure, does not formally account for potential correlation that exists between adjacent location sample points in time and space. To the extent possible, we addressed this issue through the use of an analytical script that involved randomized resampling from our experimental data. For subsequent analysis, development of an analytical method that incorporates hierarchical modeling, including both fixed and random effects, into the procedure may help address impacts of possible correlated data structures associated with real-time GNSS.

Applications of real-time positioning for logging safety need to account for the reality that both mature and clearcut conditions, and associated impacts on GNSS accuracy, occur over the course of most conventional harvesting operations in the northwestern U.S. When a harvest unit has been felled in its entirety and the rigging crew is working in the open, more accurate positioning is possible. However, higher errors should be expected for GNSS-RF applications related to manual fallers or feller-bunchers, or in partial harvesting operations, such as the John Lewis Pole cedar pole harvest. Lower accuracies attributed to canopy cover may also be compounded by terrain effects, which can reduce satellite fix rates in forested areas, particularly in valleys [28,29,34]. For example, GNSS accuracies of devices associated with the rigging crew could vary between hookers working downhill and chasers working closer to ridgelines.

If sub-meter accuracy is desired under canopies, similar to precision forestry applications that require accurate marking of skid trails or individual trees [35], ground based augmentation systems (GBAS) may be necessary. GBAS determine the degree of error and transmit corrections to rover units which can then re-calculate their positions accordingly [19]. Haughlin et al. recently achieved 0.94-m accuracy on a harvester using RTK (real-time kinematic) correction, compared to 7 m with GNSS alone [20]. It is also important to note that the Atlas PT transponders used in this study relied on only the United States’ NAVSTAR GPS constellation for position determination. Many current GNSS devices, including even consumer-grade handheld units for recreational use, are multi-constellation devices that determine position using not only GPS, but also the Russian Global Navigation Satellite System (GLONASS). Emerging devices will soon also utilize European (Galileo) and Chinese (BeiDou) navigational satellite systems as well. It is likely that newer GNSS-RF transponders capable of multi-constellation positioning will have higher accuracy in forested, mountainous locations where the number of trackable satellites may be diminished. However, use of multi-constellation sensors will not eliminate the multipath error endemic to highly reflective environments such as forests [32]. Similarly, although GBAS can greatly reduce GNSS positioning errors under canopies, differential correction cannot account for multipath effects. Even DGPS receivers will demonstrate higher errors in mature stands than in clearcuts.

According to our distance-based definitions of safe and hazardous work areas, the rigging crews we evaluated spent, on average, over one-third of their work day in unsafe conditions associated with the loader and carriage and nearly half of the day near snags. The simulated proportions of time spent in unsafe zones based on expected mature stand error varied significantly from observed proportions for six of nine tests; thus, using the technology evaluated in our study, accuracy errors associated with GNSS-RF devices under the canopy do impact perceptions of safety on logging operations, even when using basic, dichotomous definitions based on presence inside or outside a geofence. Devices with greater accuracy capabilities, at least through multi-constellation GNSS processing, and preferably RTK or other improved localization, are recommended for fine-resolution applications such as worker positioning around the landing. Proportions of safe and unsafe time differed significantly between observed and clearcut data in only one test, indicating that the higher accuracies achievable in clearcut conditions enable greater reliability in geofence alerts.

Use of GNSS-RF technology for safety applications on logging operations should be proportional to accuracy limitations. Given the large GNSS error observed under mature forest canopy in our designed experiment, single-constellation GNSS-RF radios such as the Raveon Atlas PT should only be deployed for very coarse monitoring of worker locations to improve general situational awareness and communication in forested environments; no operator decisions should be made based on observed, transmitted locations indicating the proximity of workers to jobsite hazards. That said, our operational sampling results offer a glimpse into the novel sorts of analyses that are becoming possible with real-time, networked positioning solutions in operational forestry. There is tremendous potential for improving both the safety and efficiency of logging through analysis of the high resolution spatial and temporal data that results from deployment of GNSS-RF and similar location-based services in production forestry.

Future research on GNSS-RF use for logging safety may wish to consider both vertical and horizontal positioning to better account for overhead hazards, such as the carriage, and to better specify inter-element distances on steep slopes. Future studies may also address how current positioning devices and systems can be adapted specifically for forestry applications, such as improvements to the user interface that allow loggers to utilize the technology easily and effectively with little distraction to normal work flow. This could entail display and sound settings or possible integration with other forms of data acquisition. For instance, Light Detecting and Ranging (LiDAR) information collected on snag locations could be synchronized with GNSS data to note worker proximity to snags or other environmental hazards [36]. Safety applications could also incorporate a more fluid warning system, such as through a series of proximity alerts that indicate increasing levels of danger associated with proximity to one or more hazards.

## 5. Conclusions

Atlas PT GNSS-RF positioning accuracy using only the NAVSTAR GPS system was more than three times greater in clearcut harvest units than under mature forest canopies. Error associated with mature overstory significantly affected the perception of worker safe or unsafe proximity to situational hazards. Ground workers spend approximately one-third of their time within areas of increased risk adjacent to mobile hazards such as the loader and carriage. Multi-constellation GNSS processing technology or other methods to improve localization accuracy are needed to provide the level of positioning detail necessary to avoid accidents with these fast-moving, dynamic hazards. In clearcut conditions, where errors are generally under 4 m, differential correction or other improved localization may be less critical but still recommended, especially for positioning at the landing and along the chute below the yarder.

## Figures and Tables

**Table 1.** Controlled experiment: University of Idaho Experimental Forest stand characteristics and sampling conditions.

Stand	Cover	Mean Height (m)	Mean DBH (cm)	Azimith (°)	Slope (%)	Date	Mean Satellites	Mean PDOP
260	Mature	23.52	33	95	37	10/12/16	7	1.6
58	Clearcut	NA	NA	352	18	10/17/16	8	1.3
531	Clearcut	NA	NA	165	35	10/19/16	8	1.4
290	Mature	14.6	25	35	5	10/19/16	7	1.5
345	Clearcut	NA	NA	130	8	10/24/16	10	1.3
139	Mature	16.7	31	347	43	10/24/16	6	1.6
524	Mature	17.3	31	27	14	11/10/16	6	1.7
262	Clearcut	NA	NA	205	2	11/17/16	8	1.2

**Table 2.** Root mean square error (RMSE) of Atlas PT GNSS horizontal positioning error, in meters, at each unit position (A, B, C, and D) and across all units (last column).

Cover	Stand	Unit RMSE (m)				
		A	B	C	D	All Units
MATURE	260	13.07	8.29	12.52	4.79	10.34
	290	11.68	6.91	7.48	7.28	8.56
	139	11.23	7.68	12.78	7.90	10.14
	524	18.17	11.16	11.59	15.30	14.34
CLEARCUT	58	4.33	5.36	2.98	1.46	3.81
	531	2.42	1.67	3.16	3.01	2.64
	345	1.38	1.49	3.09	3.66	2.67
	262	3.96	3.69	4.52	4.07	4.09

**Table 3.** Proportion of instances (1-second intervals) when ground workers occupied unsafe zones associated with each hazard based on observed, mature, and clearcut data, with sample size, n, shown in parentheses.

	Loader			Carriage			Snag		
Site	Obs.	Mat.	Clear.	Obs.	Mat.	Clear.	Obs.	Mat.	Clear.
JLP	0.404	0.382 (*n* = 100,886)	0.406	0.252	0.238 (*n* = 72,251)	0.257	0.503	0.497 (*n* = 110,495)	0.512
WTS	0.226	0.220 (*n* = 66,872)	0.225	0.477	0.449 (*n* = 70,430)	0.477	0.363	0.361 (*n* = 76,926)	0.362
UH	0.492	0.487 (*n* = 21,742)	0.491	0.449	0.423 (*n* = 56,490)	0.449	0.528	0.498 (*n* = 61,826)	0.523

**Table 4.** Results of the Marascuillo Procedure, comparing unsafe proportions across hazards and sites (alpha = 0.05).

Test	Compared Proportions	Value (Test Statistic)	Critical Range	Value > Critical Range?
1	JLP_L_-JLP_C_	0.152	0.009	yes
2	JLP_L_-JLP_S_	0.099	0.008	yes
3	JLP_L_-WTS_L_	0.178	0.009	yes
4	JLP_L_-UH_L_	0.088	0.015	yes
5	JLP_C_-JLP_S_	0.251	0.009	yes
6	JLP_C_-WTS_C_	0.225	0.01	yes
7	JLP_C_-UH_C_	0.197	0.01	yes
8	JLP_S_-WTS_S_	0.14	0.009	yes
9	JLP_S_-UH_S_	0.025	0.01	yes
10	WTS_L_-WTS_C_	0.251	0.01	yes
11	WTS_L_-WTS_S_	0.137	0.009	yes
12	WTS_L_-UH_L_	0.266	0.015	yes
13	WTS_C_-WTS_S_	0.114	0.01	yes
14	WTS_C_-UH_C_	0.028	0.011	yes
15	WTS_S_-UH_S_	0.165	0.01	yes
16	UH_L_-UH_C_	0.043	0.016	yes
17	UH_L_-UH_S_	0.036	0.016	yes
18	UH_C_-UH_S_	0.079	0.011	yes

**Table 5.** Results of the Marascuillo Procedure, comparing unsafe proportions of observed, mature, and clearcut data (alpha = 0.05).

Test	Compared Proportions	Mean Value (Test Statistic)	Mean Critical Range	Value > Critical Range?
JLP Loader	Obs.-Mat.	0.022	0.005	yes
	Obs.-Clear.	0.002	0.005	no
	Mat.-Clear.	0.024	0.005	yes
JLP Carriage	Obs.-Mat.	0.014	0.006	yes
	Obs.-Clear.	0.005	0.006	no
	Mat.-Clear.	0.019	0.006	yes
JLP Snag	Obs.-Mat.	0.007	0.005	yes
	Obs.-Clear.	0.009	0.005	yes
	Mat.-Clear.	0.016	0.005	yes
WTS Loader	Obs.-Mat.	0.006	0.006	no
	Obs.-Clear.	0.001	0.006	no
	Mat.-Clear.	0.005	0.006	no
WTS Carriage	Obs.-Mat.	0.027	0.007	yes
	Obs.-Clear.	0.000	0.007	no
	Mat.-Clear.	0.027	0.007	yes
WTS Snag	Obs.-Mat.	0.002	0.006	no
	Obs.-Clear.	0.000	0.006	no
	Mat.-Clear.	0.002	0.006	no
UH Loader	Obs.-Mat.	0.005	0.012	no
	Obs.-Clear.	0.001	0.012	no
	Mat.-Clear.	0.004	0.012	no
UH Carriage	Obs.-Mat.	0.026	0.007	yes
	Obs.-Clear.	0.001	0.007	no
	Mat.-Clear.	0.027	0.007	yes
UH Snag	Obs.-Mat.	0.030	0.007	yes
	Obs.-Clear.	0.005	0.007	no
	Mat.-Clear.	0.024	0.007	yes
